# Mastopexy using de-epithelialised dermoglandular flaps: a case series for maximal volume conservation following breast implants removal

**DOI:** 10.1186/s40001-022-00790-0

**Published:** 2022-08-27

**Authors:** Umar Daraz Khan, Salma Naseem, Sadia Rafiq

**Affiliations:** 1 Reshape Clinic, Reshape House, 2-4 High Street, West Malling, Kent, ME19 6QR UK; 2 Yeovil District Hospital, Yeovil. Somerset, BA21 4AT UK; 3 Department of Plastic and Reconstructive Surgery, St George’s Hospital, London, SW1 0QT UK

**Keywords:** Explantation surgery, Mastopexy, Dermoglandular flaps, Autoaugmentation, Bostwick flap

## Abstract

**Aims and objective:**

Removal of implants without replacement is often requested, and the procedure is more commonly performed today than ever before. However, the resultant loss of body image, secondary to the loss of breast volume, is not an outcome, that a patient is looking forward to. There is a lack of information on the options available to the patients following explantation. This case series presents an option of breast volume preservation and reshaping during mastopexy after breast implant removal that can be offered to selected patients. In the current case series, de-epithelialised dermoglandular flap mastopexy was used as an autologous tissue for breast reshaping and remodelling.

**Material and methods:**

Since 2015, ten patients were selected for de-epithelialised dermoglandular mastopexy using wise pattern or vertical scar. Surgery was performed under general anaesthesia as a day case. A vertically oriented bipedicular dermoglandular flap was used for vertical scar mastopexy in two patients, and eight patients had Wise pattern incisions. Of these eight patients, four had superomedial and four had inferiorly based flaps for dermoglandular mastopexy and closure. All patients had a preoperative cup size D or larger.

**Results:**

All patients had adequate results with an acceptable breast cup size. There was no skin breakdown, nipple loss, haematoma or infection.

**Conclusion:**

De-epithelialised dermoglandular flap mastopexy is a safe procedure and can be used as an option in selected patients.

**Level of Evidence:**

IV.

## Introduction

Since the introduction of preformed breast implants, augmentation mammoplasty has been a commonly performed procedure [1]. Techniques that utilise various pockets for implant placements have been described with acceptable results [2]. However, when an implant is placed in a limited and confined space, the noncompressible prosthesis exerts pressure on the compressible skin envelope, which inevitably results in tissue compression and skin envelope thinning over time. The instant expansion of the envelope followed by tissue thinning and subsequent weight, volume and related forces stretch the breast skin envelope. The stretching effects are time, weight, volume and breast implant pocket-dependent. For implants of the same size and over the same period of time, tissue stretching is seen more in the subglandular pocket than in the submuscular pocket and heavier implants cause more stretching regardless of the pocket used [3, 4]. When explantation of the breast implants is performed without replacement, the procedure leaves an empty and stretched skin (tissue) envelope, the size of which depends on the size of the implant, the duration since the first operation and the implant pocket. Weight or pregnancy related changes in the breast during this time might also have an impact on the size of the breast, the quality of the skin envelope and the positioning of the nipple. Therefore, it is no surprise that these breasts are often ptotic and quite frequently require mastopexy following explantation. Routinely available mastopexy procedures are challenging in these cases and the resultant skin and tissue resections may further compromise the breast cup size.

One-stage mastopexy-lipofilling after breast implant removal remains a good option following explantation in patients lacking breast tissue for adequate breast volume restoration [5, 6]. However, patients with a history of generalised weight gain, gain in breast volume following augmentation mammoplasty, feel that they are too heavy on chest, get their implants removed due to the risks of breast implant-associated anaplastic large cell lymphoma (BIA-ALCL) or are no longer interested in continuing having breast implants, dermoglandular flaps provide an addition to the current armamentarium of limited options available. The current article is a case series of 10 consecutive selected patients who were offered the de-epithelialised dermoglandular flap technique with vertical or Wise pattern scars for breast volume management.

## Material and methods

A retrospective analysis was carried out on data from de-epithelialised dermoglandular flaps for mastopexies following breast implant removal performed between October 2015 and October 2021.

## Clinical history

Meticulous medical and clinical history is paramount. This study evaluated the duration since the augmentation mammoplasty, breast cup size prior to breast augmentation, size of the implants, postmammoplasty breast cup size and premastopexy breast cup size at the time of consultation (Tables 1 and 2). Any history of previous or current breast or chest asymmetry was an important aspect of the patient’s history and is noted. The presence or absence of capsular contracture, breast lumps and axillary lymph node status was also recorded. The breast cup sizes were measured using the traditional Zheng method [7]. The size of the implant used is absolutely essential for determining the possible final breast cup size. Regnault previously showed that approximately 100 cc is required to increase the circumference of the breast by 1 inch, which is one increase in cup size [8]. The end cup size that would result from the procedure was predicted from the current cup size measured using the Zheng technique minus the implant volume.

**Table 1:** The generalised distribution of premammoplasty and postmammoplasty measurements and characteristics

	Age	Time since first operation	Pre-op cup size	Implant size (cc)	Post-op-cup size	Implant surface	Implant pocket	Capsular contracture
1	42	5	34B	450	34E	Textured	Subglandular	Rt I, Lt I
2	49	14	34B	230right 300 left	34D	Textured	Muscle splitting biplane	Rt I, Lt I
3	47	11	36C	300	36DD	Textured	Muscle splitting biplane	Rt I, Lt IV
4	55	10	36B	390LT, 460RT	36DD	Textured	Muscle splitting biplane	Rt III, Lt I
5	61	19	34B	325	34DD	Textured	Subglandular	III, IV
6	48	8	34B	325	34DD	Textured	Subglandular	III, III
7	50	8	34b	325	34DD	Textured	Subglandular	III, III
8	38	7	34B	460	34E/EE	Textured	Muscle splitting biplane	I, I
9	36	16	34 A	265	34 C	Textured	Submuscular	I, I
10	56	12	36 B	440	36DD	Textured	Subglandular	I, I

**Table 2:** The relative distribution of premammoplasty and postmammoplasty breast cup sizes in the series

Cup size	Premammoplasty	Postmammoplasty
34A	1 (10%)	–
34B	6 (60%)	–
34C	–	1 (10%)
34D	–	4 (40%)
34E	–	2 (20%)
36B	2 (20%)	–
36C	1 (10%)	–
36D	–	3 (30%)

### Inclusion criteria

Patients who presented with a premastopexy cup size of DD, with at least two breast cup sizes gained following their augmentation mammoplasty, were offered the dermoglandular technique.

### Markings and technique

Breast marking was performed in the standing position. Measurements of the current cup size, suprasternal notch (STN) to nipple areolar complex (NAC), and NAC to inframammary crease (IMC) were taken (Table 3).

**Table 3:** The generalised distribution of premastopexy and postmastopexy measurements and characteristics

	Premastopexy cup	Ptosis	Preoperative sternal notch to NA (cm)	Preoperative NAC to IMC (cm)	Markings	Flap design	Postmastopexy cup	Postoperative STN to NAC, Rt, Lt (cm)	Postoperative NAC to IMC Rt, Lt (cm)
1	34E	III,III	26,26	11	Wise pattern	Superomedial	34C	22,22	8.5,8.5
2	34DD		29,25	11,11	Wise pattern	Superomedial	34C	22,22	8,8
3	34EE	N,N	23,23	12,12	Vertical scar	Bipedicle	34C	21,21	10,10
4	36 DD/E	N,N	24,25	9,9	Wise pattern	Inferiorly based	36 C	20,20	7,7
5	34DD	III,III	26,26	9,9	Wise pattern	Superomedial	34 C	22,22	8,8
6	34DD	III,III	25,24.5	13,13	Wise pattern	Inferiorly based	34C	21,20	7,7
7	34DD	III,III	25,25	13,13	Wise pattern	Inferiorly based	34 C	21,20	7,7
8	34FF	II,II	24,25	10,10	Wise pattern	Medially based	34D	22,22	7.5,7.5
9	36 DD	I,I	24, 25	9.5,9	Vertical scar	Bipedicled	36 C	22,22	8.5, 8.5
10	36 EE	N,N	24,23	11,12	Wise pattern	Inferiorly based	36 B	20.5,20.5	6.5,6.5

STN; suprasternal notch, NAC; nipple areolar complex, IMC; inframammary crease

**Fig. 1:**
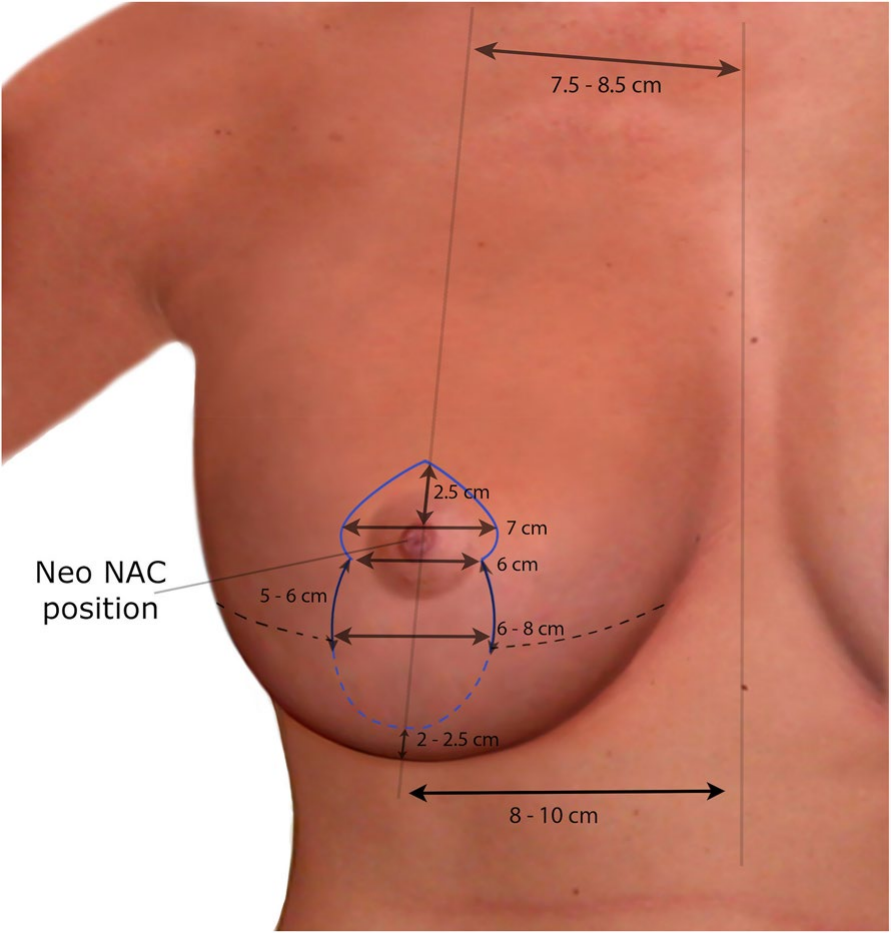
Illustration showing hybrid markings for both vertical and wise pattern incisions

Markings for mastopexy were selected on the basis of the NAC to IMC distance. Vertical scarring was used if the preoperative NAC to IMC distance was less than 8 cm and Wise pattern marking was used if the distance was 9 cm or more. The IMC was marked all along its width, and a midline was marked between the STN and xiphisternum. The neo-NAC was marked on the breast meridian line, using the IMC as the reference and at 1 cm higher than the IMC projection on the breast. A keyhole was drawn with its upper limit, 2.5 cm above the new nipple position with a width of 7 cm at its widest point and 5 to 6 cm at the neck of the keyhole. From the neck of the keyhole, two lines were drawn down and away, each 5 to 6 cm long on average and usually not more than 6 to 8 cm apart. For vertical scar markings, the two lines continued down toward a point 2.5 cm higher than the marked IMC. In the Wise pattern markings, the medial and lateral markings were extended to meet the IMC at its respective end (Fig. 1). These markings were always checked and adjusted as necessary before and after explantation for safe and tension-free closure.

**Fig. 2:**
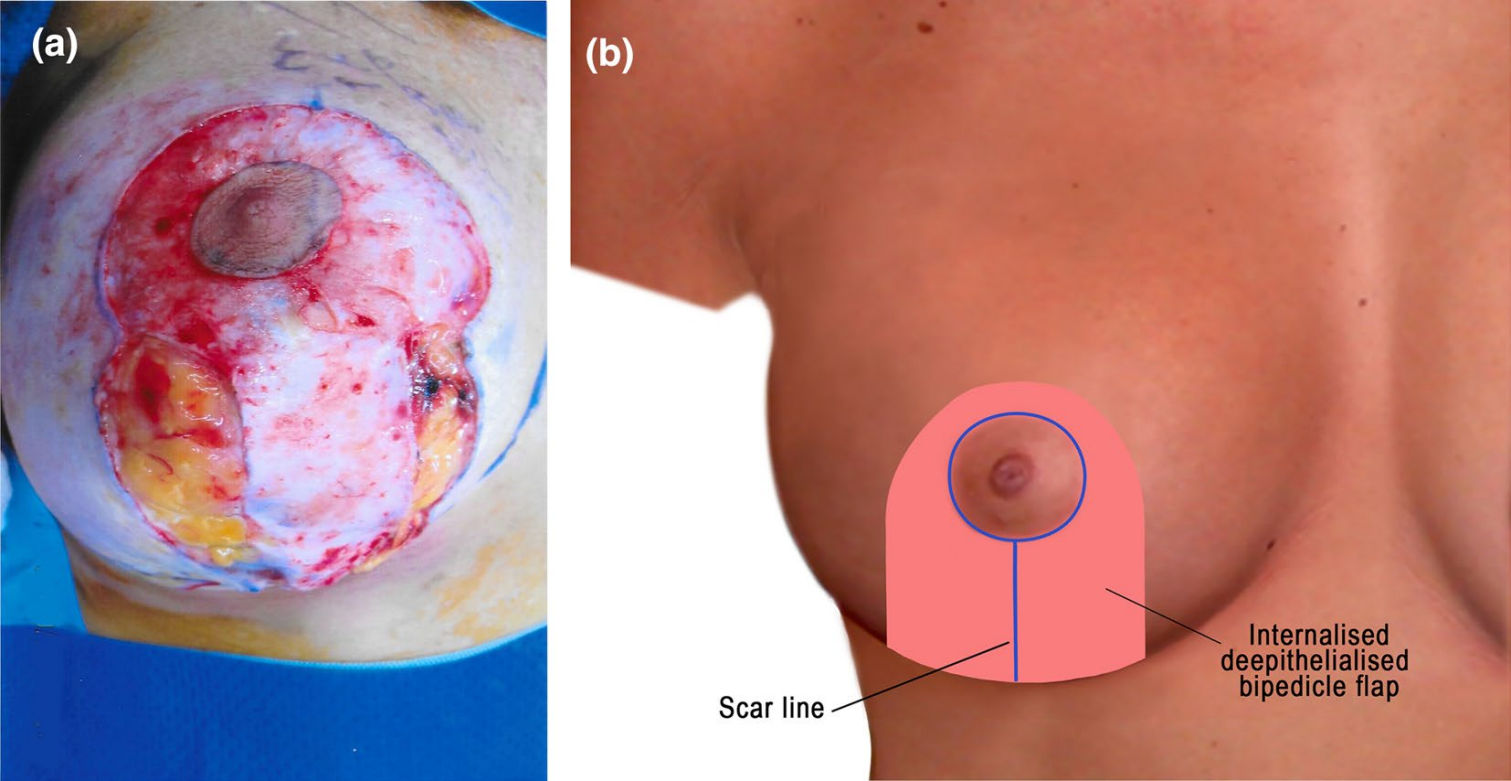
**a** A vertically oriented bipedicle de-epithelialised dermoglandular flap with 4.2 cm NAC. The de-epithelialised bipedicle flap is incised, from the neck of the keyhole down to the inferior limits of the markings. **b** Illustration showing appearance following explantation and closure in layers. The pink-shaded area is the internalised de-epithelialised vertical bipedicle flap

A 4.2-cm nipple marker was used to mark the neo-NAC. In the vertical scarring cases, the rest of the skin was de-epithelialised (Fig. 2a). The dermis and gland were incised starting from the neck of the keyhole, which produced a vertically oriented bipedicle flap (Fig. 2a). Complete capsulectomy is performed in Grade III/IV capsules presenting in the subglandular pocket and complete or near total capsulectomy is performed in submuscular pockets. Thick capsules are sent for histopathology, and where necessary or excess fluid was present, samples were taken for CD30 analysis [9].

**Fig. 3:**
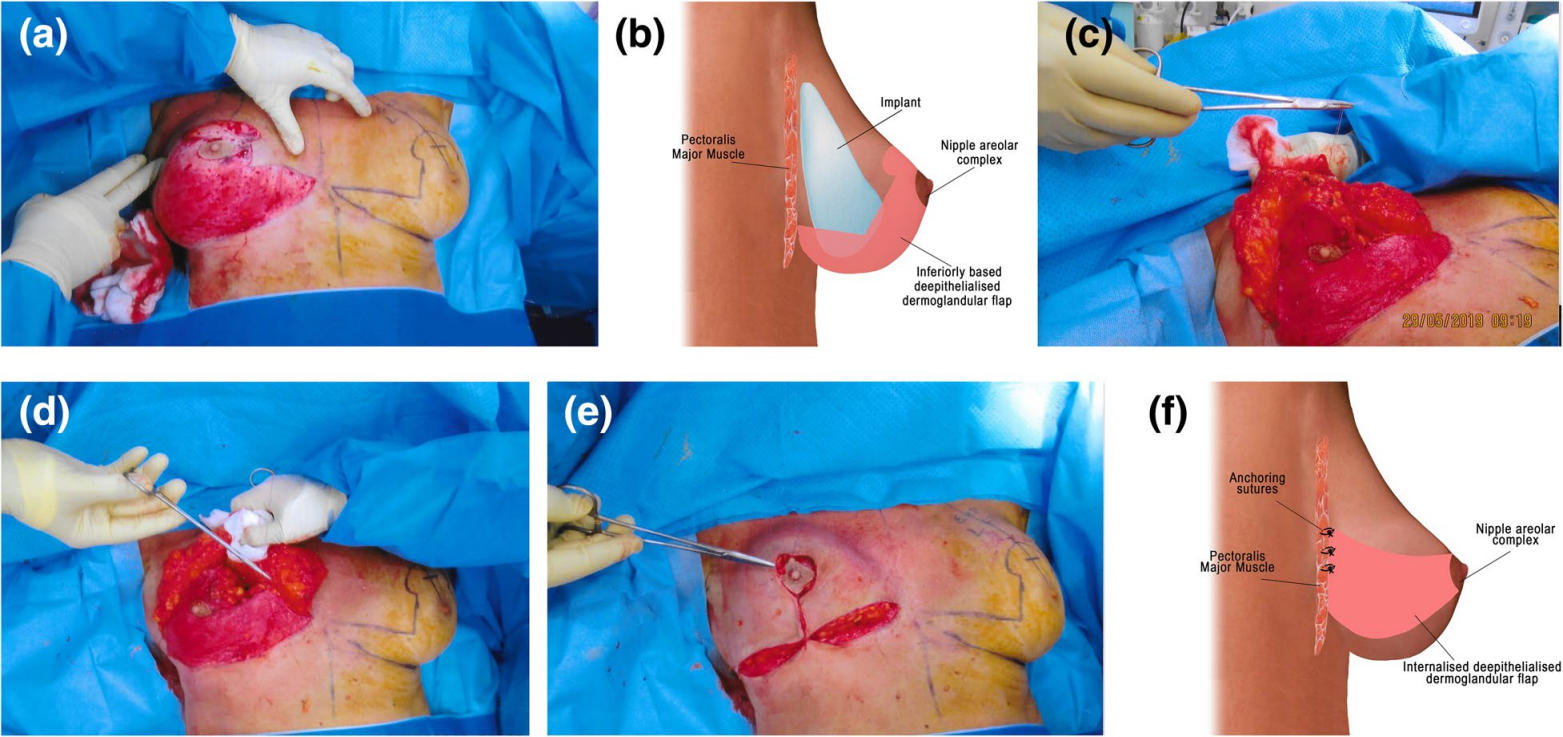
**a** Patient in the supine position with Wise pattern markings. The whole extent of the markings is de-epithelialised for the inferior dermoglandular flap. **b** Illustration showing a lateral view of an inferiorly based de-epithelialised dermoglandular flap with implants in situ. **c**, **d** An inferiorly based flap on the right breast following explantation. Full thickness inferior dermoglandular flap is incised and partially sutured to the pectoralis major, all along its extensions. **e**–**f** Illustration showing inverted T closure following internalisation and closure of the inferiorly based dermoglandular flap

For Wise pattern marking cases, the NAC pedicle orientation was selected based on the difference between the preoperative STN to NAC distance and the STN to the newly marked NAC distance. If the difference between these two measurements was more than 6 cm, then the whole extent of the marked skin was de-epithelialised for an inferiorly based flap and NAC pedicle circulation safety (Fig. 3a−b). If the difference between these two measurements was less than 6 cm, then a superomedial flap was selected.

When a superomedial-based flap was selected, a de-epithelialised dermoglandular flap was incised horizontally below the level of the superomedial flap and along the lateral and medial extension, and its superior margin was sutured to the pectoralis muscle all along using 2-0 Vicryl. When an inferiorly based flap was used, the whole dermoglandular flap was incised along its markings and sutured to the chest wall, except for the flap’s middle section around the NAC, which remained unstitched to the chest wall. This allowed for better NAC pedicle mobilisation (Fig. 3c−f).

In patients who had implants of two different sizes, which were used for breast asymmetry, excess breast tissue, equivalent to the difference in the implant volumes, was excised from the larger breast (with the smaller implant). Drains are used where total capsulectomy is performed in a thickened capsule. Closure was performed in layers using 2-0 Vicryl for the medial and lateral pillars and 3-0 Vicryl and 4-0 Monocryl for subcutaneous and intradermal closure, respectively (Fig. 2b).

## Statistical analysis

The data were analysed using the Statistical Package for the Social Sciences (SPSS), version 19.0. The results are presented in the text as frequencies and percentages for qualitative/categorical variables (i.e. differences in implant size) and means ± S.D for quantitative/continuous variables (age and implant size). The Chi-square test was used to compare the categorical variables, and the *t* test was used to compare the quantitative/continuous variables. In all statistical analyses, only *p* values < 0.05 were considered significant.

## Results

**Fig. 4:**
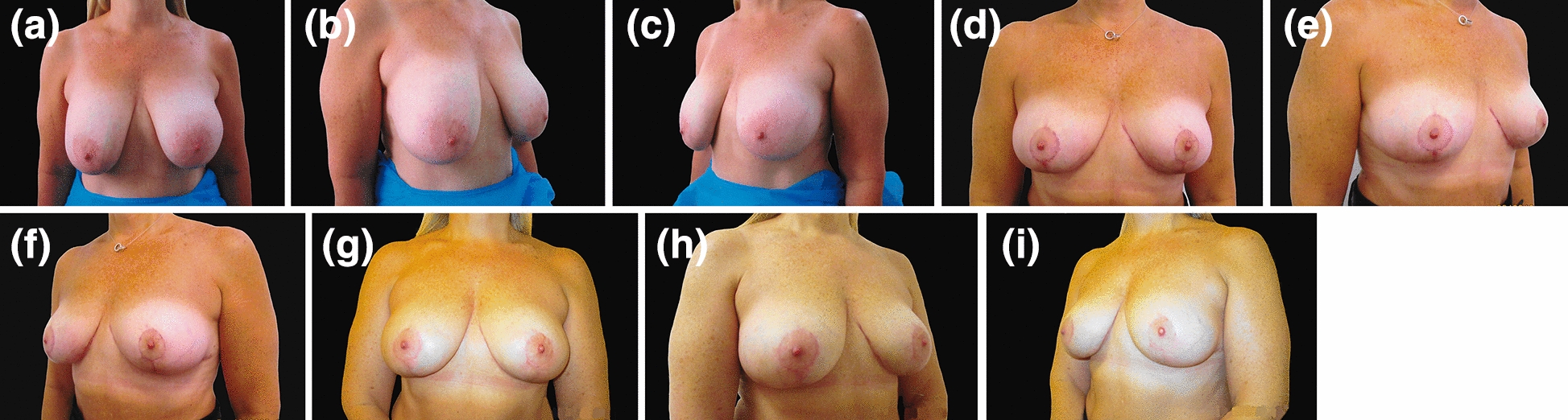
**a**–**c** Preoperative views of a 49-year-old patient who underwent breast augmentation 14 years ago using 230 and 300 cc silicone implants on her right and left sides, respectively. She presented with bilateral Regnault grade III ptosis and cup size DD. She wanted to have her implants removed without replacement. **d**–**f** 3-month postoperative views. **g**–**i** Postoperative views taken 6 months following explantation and superomedial-based inferior de-epithelialised dermoglandular flap mastopexy. She had 70 g of tissue removed from her larger right breast

**Fig. 5:**
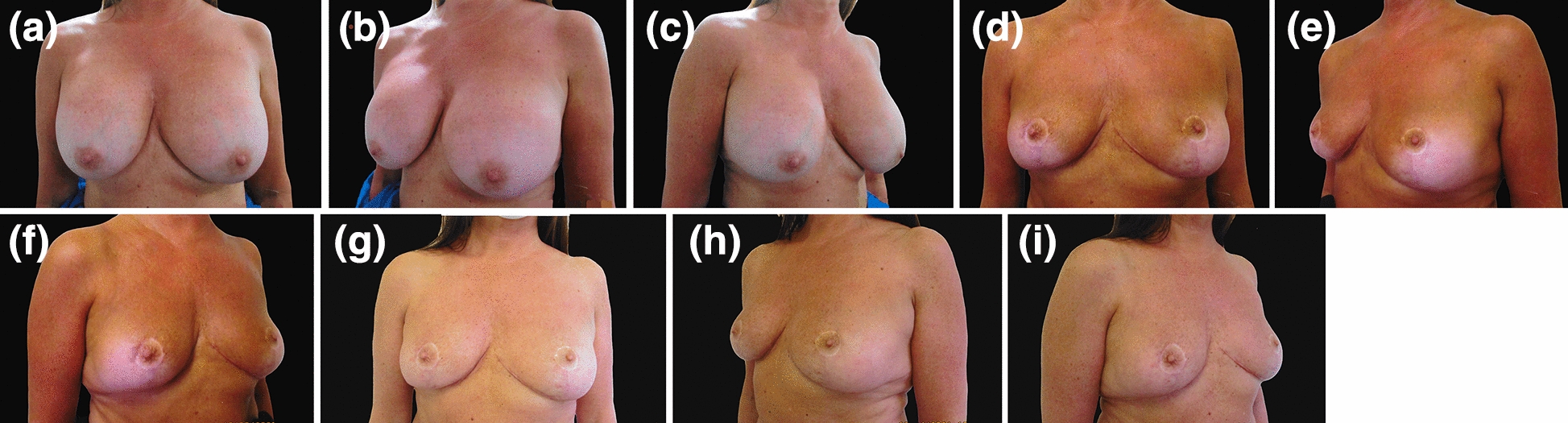
**a**–**c** Preoperative views of a 55-year-old patient who had 390 and 460 cc round cohesive gel silicone implants on her left and right side, respectively. She developed grade III capsular contracture on her right sides, respectively. She had no ptosis and presented with a 36 E breast cup size. **d**–**f** A year and 3 months following explantation and Wise pattern markings for inferiorly based de-epithelialised dermoglandular flaps mastopexy. She had a 36 C postoperative cup size. The patient developed overactive scars, which were successfully treated using intralesional triamcinolone injections. **g–i** Postoperative views taken 2 years following the surgery showed acceptable and stable results

A total of ten patients had their implants removed and opted for de-epithelialised dermoglandular flaps. The mean age of the patients was 48.2 ± 7.94 years (range 36–61 years), and the mean time from the first operation was 11 ± 4.35 years (range 5–19 years) and the mean size of the implants was 358 ± 77.5 cc. All patients had textured implants placed in muscle splitting (n = 4) subglandular (n = 5) or partial submuscular (n = 1) pockets (Table 1). Of these 10 patients, 5 had grade I capsular contracture, and the other five patients had varying degrees of capsular contracture (Table 1). Five patients had grade III ptosis, one patient had grade II ptosis, one patient had grade I ptosis, and three patients had no ptosis (Table 3). Of these ten patients, eight had a Wise pattern, and two had vertical scar markings. An inferiorly based pedicle was used in 4 patients, a superomedial pedicle was used in 4 patients, and two patients had vertical bipedicle flaps (Table 3). Premastopexy and postmastopexy STN to NAC and NAC to IMC crease measurements were recorded (Table 3). Pre-mammoplasty, postmammoplasty, premastopexy and postmastopexy cup sizes were recorded for all patients (Tables 1, 2, 3 and 4). Patients included in the series were followed up for at least six months to two years (Figs. 4 and 5). There was no postoperative ptosis recorded in the series. No patients experienced infection, wound breakdown or nipple necrosis.

**Table 4:** The relative distribution of premastopexy and postmastopexy cup sizes

Cup size	Premastopexy	Postmastopexy
34C	–	6 (60%)
34DD	4 (40%)	1 (10%)
34E	1 (10%)	–
34EE	1 (10%)	–
34FF	1 (10%)	–
36B	–	1 (10%)
36C	–	2 (20%)
36DD	2 (20%)	–
36EE	1 (10%)	–

## Discussion

Breast augmentation is one of the most common aesthetic procedures performed in the USA. In 2020 alone, 252,022 patients underwent primary breast augmentation, which was the 2^nd^ most common cosmetic surgery. In addition, 109,619 patients had their implants removed and replaced which was the 4^th^ most common breast surgery, while another 49,631 patients had their implants removed without replacement, which was the 5^th^ most common breast surgery. Breast augmentation was the most common aesthetic procedure in patients between 17 and 35 years of age. In contrast, in 2015, 305,856 patients had primary breast augmentation, and 38,071 patients had breast implants removed without replacement. There are no data for implant removal and replacement for that year. Nevertheless, breast augmentation was the second most common aesthetic procedure in 2015, while removal of implants was the 10^th^ most common procedure [10]. From the data given above, one can conclude that although there was a small decline in total breast augmentation procedures performed in 2020 compared to 2015, more patients had their implants removed without replacement. This trend may well be temporary but is likely due to recent reports of breast implant-associated anaplastic large cell lymphoma (BIA-ALCL) for textured implants [9]. In 2016, the WHO classified BIA-ALCL as a disease with an incidence rate of 1 in 24,000 implants in the UK [11]. Even though there has been a large shift towards the use of smooth implants, one can assume from the statistics that more surgeons are expected to perform explantations of breast implants without replacement. Loss of the feminine silhouette and changes in the shape or volume of the breast following an explantation without replacement may not be acceptable to the patient. The decision of explantation can be challenging because having a mastopexy associated with a skin envelope resection will further compromise the breast cup volume, while explantation alone will leave the patients with empty breasts and worsening of breast ptosis.

In oncoplastic breast-conserving surgery, localised skin flaps can be designed based on lateral and inferior perforators surrounding the breast [12]. However, an inferior-based de-epithelialised dermoglandular flap, a random pattern flap, has also been used in selected cases for implant-based breast reconstruction following mastectomy [13–15]. The nipple areolar complex (NAC) can also be used as a free nipple graft in these cases. [16] The flap allows natural, local and well vascularised autologous tissue reinforcement that improves implant safety and support following mastectomy and radiotherapy [13–16]. Following the introduction of these flaps in oncologic breast reconstruction, the idea was extended to aesthetic mastopexies with augmentation. The flap provides support and safety to the implants during wound healing, and in the long term, these flaps provide support to the lower pole to prevent bottoming-out [17–24]. The use of an inferior-based flap for projection following breast reduction has also been eloquently described by Ribeiro [25]. The use of these flaps was further advanced by Graf et al., who used them for mastopexy in conjunction with autofat grafting for breast reshaping and volume enhancement [26]. Ribeiro and Graf flaps are attached and anatomically continuous to the chest wall along their length with NAC attached in contrast, in dermoglandular flaps, earlier implant pocket dissection degloves the skin envelope almost in its entirety and blood supply to the whole skin, including NAC, solely depends on thin peripheral margins of the skin envelope. The use of this inferior-based de-epithelialised dermoglandular flap for autologous tissue breast remodelling was recently described by the senior author of this paper [27].

Simple explantation without replacement is an option however, de-epithelialised dermoglandular flap Wise pattern or bipedicle vertical scar mastopexy is an option frequently discussed with qualifying patients [14, 27–29]. Further breast volume enhancements, if required, can be contemplated via autologous fat grafting, as shown in a series by Mangialardi ML et al. [5, 6]. The concept of autoaugmentation is not new and was described by Graf and Biggs for mastopexy and reduction as a de-epithelialised inferiorly based flap passed through a loop of pectoralis major muscle [30]. Lipofilling on its own is routinely used for breast augmentation and when used as an adjunct to mastopexy, obviates the need for breast implants [5, 26, 31]. The measurement of the breast cup size using the Zheng method and a 100 cc volume equivalent for every one inch increment in breast circumference by one inch has been challenged due to its inaccuracy [7, 8, 32]. However, the results in this limited study show that overall, the two methods were fairly predictable in terms of their accuracy. Ideally, the use of MRI or 3D digital photography for breast volume measurements would provide more accurate volume and cup size predictions. All but one patient in this case series had a breast cup size of C or larger after implant removal (Tables 3 and 4). The choice of these flaps should ideally be a process of informed consent to alleviate the worries of these patients over the loss of body image following explantation surgery. Even though most of the patients were happy with the outcome, no assessment of patient satisfaction was included in this limited study.

### Weaknesses of the study

This is a small case series with short-term results using different types of dermoglandular flaps and NAC flap orientations. A larger cohort of patients in a long-term follow-up study is needed to evaluate the stability and durability of the volume and the results of these techniques. Even though the first case was reported six years ago, most of the cases were performed just prior to the pandemic and therefore lacked a long-term follow-up due to pandemic-related restrictions. Another weakness in the study is that most of the patients had gained considerable volume in breast cup size following their initial augmentation mammoplasty due to generalised weight gain. However, it was difficult for patients to remember information about their weight prior to their augmentation mammoplasty, therefore it was not included in this study. Patient outcome analysis of the results was not performed using standardised questionnaires such as the BREAST Q or SF-36 or a PROMIS score.

## Conclusion

The use and safety of these flaps in our small sample demonstrate that inferior dermoglandular flaps can be a useful option in selected patients, as specified above, who are considering breast implant removal without prosthesis replacement.

## Data Availability

The datasets used and/or analysed during the current study are available from the corresponding author on reasonable request.
